# A Systematic Review of Hospital-to-School Reintegration Interventions for Children and Youth with Acquired Brain Injury

**DOI:** 10.1371/journal.pone.0124679

**Published:** 2015-04-29

**Authors:** Sally Lindsay, Laura R. Hartman, Nick Reed, Caron Gan, Nicole Thomson, Beverely Solomon

**Affiliations:** 1 Bloorview Research Institute, Holland Bloorview Kids Rehabilitation Hospital, Toronto, Ontario Canada; 2 Department of Occupational Science & Occupational Therapy, University of Toronto, Toronto, Ontario, Canada; 3 Bridgepoint Active Healthcare, Toronto, Ontario, Canada; 4 Brain Injury Rehabilitation Outpatient Team, Holland Bloorview Kids Rehabilitation Hospital, Toronto, Ontario, Canada; Institute of Automation, Chinese Academy of Sciences, CHINA

## Abstract

**Objectives:**

We reviewed the literature on interventions that aimed to improve hospital-to-school reintegration for children and youth with acquired brain injury (ABI). ABI is the leading cause of disability among children and youth. A successful hospital-to-school reintegration process is essential to the rehabilitative process. However, little is known about the effective components of of such interventions.

**Methods and findings:**

Our research team conducted a systematic review, completing comprehensive searches of seven databases and selected reference lists for relevant articles published in a peer-reviewed journal between 1989 and June 2014. We selected articles for inclusion that report on studies involving: a clinical population with ABI; sample had an average age of 20 years or younger; an intentional structured intervention affecting hospital-to-school transitions or related components; an experimental design; and a statistically evaluated health outcome. Two independent reviewers applied our inclusion criteria, extracted data, and rated study quality. A meta-analysis was not feasible due to the heterogeneity of the studies reported. Of the 6933 articles identified in our initial search, 17 articles (reporting on 350 preadolescents and adolescents, aged 4–19, (average age 11.5 years, SD: 2.21) met our inclusion criteria. They reported on interventions varying in number of sessions (one to 119) and session length (20 minutes to 4 hours). The majority of interventions involved multiple one-to-one sessions conducted by a trained clinician or educator, homework activities, and parental involvement. The interventions were delivered through different settings and media, including hospitals, schools, and online. Although outcomes varied (with effect sizes ranging from small to large), 14 of the articles reported at least one significant improvement in cognitive, social, psychological, or behavioral functioning or knowledge of ABI.

**Conclusions:**

Cognitive, behavioral, and problem-solving interventions have the potential to improve school reintegration for youth with ABI. However, more comprehensive interventions are needed to help link rehabilitation clinicians, educators, adolescents, and families.

## Introduction

Acquired Brain Injury (ABI) is the leading cause of death and disability among children [1–2] and a significant public health concern [3]. It is defined as damage to the brain that occurs after birth from a traumatic (e.g., blow to the head, fall, motor vehicle accident, sports injury) or non-traumatic (e.g., illness, stroke) event [4–6]. In the United States, pediatric ABI results in approximately 475,000 emergency department visits and 37,000 hospitalizations each year [2, 7], costing the health care system up to $60 billion annually [8–9]. Given the economic and social benefits of reducing the length of hospital stays, health care providers are increasingly sending children and youth from hospitals to home and school environments in the midst of ongoing recovery [10]. Greater responsibility for care is being placed on children and youth, families, and educators, following hospitalization [11]. However, the readiness of children and youth to return to normal activities and their adherence to self-management regimes may be less than optimal [10, 12–14]. Children (defined as 2–13 years old) and youth (defined as 14–19 years old) with ABI can continue to experience neurological changes for weeks, months, or years following reintegration to home and school environments [15].

The ABI recovery patterns of children and youth differ from those of adults. Their brains continue to develop well into young adulthood, and latent brain injury-related challenges may not become readily apparent until children enter adolescence [15]. After a mild traumatic brain injury, one is at a greater risk for a second brain injury [16]. Youth with brain injuries are also three times more likely to attempt suicide and roughly twice as likely to be bullied at school or online, to bully others, to seek help from a crisis help line, or to be prescribed medication for depression, anxiety, or both [17].

Poor transitions from hospital to school environments can negatively influence health outcomes (i.e., physical, psychological, and cognitive functioning) and meaningful participation in life [5, 18–20]. Effective hospital-to-school reintegration programs can help mitigate those risks. They aim to improve the capacity of children and youth to maintain their physical and emotional health; as such, they can be considered a form of health promotion and secondary prevention. They are a key component of the rehabilitation process [21–22] and have been linked to improved health outcomes (i.e., improved neuropsychological functioning, problem-solving, social competence, and independence; reduced anxiety, depression, and withdrawal symptoms) and reduced re-hospitalization [2, 13, 23–30].

Recent evidence suggests the design and delivery of effective transition programs are critical for effective pediatric rehabilitation [31]. However, standardized practices in hospital-to-school reintegration programs are currently lacking. Given the substantial variation in their delivery (e.g., how, when, where, and by whom they are delivered) [22, 32], it is critical to identify best practices and effective components (e.g., structure, organization, and specific processes) of hospital-to-school transition programs [22].

Currently, several gaps exist in the research on ABI transition interventions. First, most reviews of ABI intervention research have focused on adults [33]. Furthermore, studies addressing transitions among children and youth have focused on community integration more broadly, rather school environments specifically. Second, little is known about the types of interventions that are appropriate for particular developmental stages and severity levels of ABI, as well as the delivery methods, contexts, and cost-effectiveness involved. Finally, a better understanding is needed of the theoretical foundations (e.g., health promotion strategies, theories of behavior change) underlying efforts to promote and evaluate positive health outcomes among children and youth with ABI.

In this systematic review, we aim to inform the development of effective hospital-to-school reintegration programs. Specifically, our objectives are to: (1) critically appraise and synthesize best practices and effective components (i.e., content, length, format, delivery site, delivery method) of hospital-to-school reintegration interventions focusing on the physical and psychosocial health of children and youth with ABI; (2) develop key recommendations to inform standards of care for pediatric transitions for youth with ABI to enhance health-related knowledge among of youth and their caregivers; and (3) highlight gaps in understanding and areas for future research.

## Methods

### Search strategy and data sources

Our research team conducted a comprehensive search of peer-reviewed published literature using health-related databases, including: Ovid MEDLINE, HealthSTAR, Cumulative Index to Nursing and Allied Health Literature (CINAHL), Cochrane Database of Systematic Reviews, EMBASE, ERIC, and PsycInfo (Table 1). We searched for subject headings and MeSH terms related to hospital-to-school transitions and ABI, including: “hospitalized children”, “transitional programs”, “transition process”, “hospital-to-school transition”, “school health services”, “school re-entry”, “school re-integration”, “school liaison”, “hospital classrooms”, “special education”, “discharge planning”, “rehabilitation”, “intervention”, “program planning / evaluation”, “brain injury”, “craniocerebral trauma”, “concussion”, “brain neoplasms”, “head injury”, “brain tumor”, “stroke”, and “cerebrovascular accident.” We also implemented database restrictions related to age (child, youth, adolescent, young adult). We used a range of terms reflecting experimental design (e.g., randomized controlled trial (RCT), before-and-after design) because we recognized the methodological diversity and challenges presented by this literature and wanted to avoid overlooking relevant interventions. We mapped each term to the subject heading using “auto explode” and advanced search options. We searched for articles published between January 1989 and June 2014. We implemented no language restrictions at the time of the search. We made minor modifications to our search as needed for individual databases (see Table 2 for Ovid MEDLINE). We also manually examined the reference lists of all articles selected for review to identify additional articles for inclusion.

**Table 1:** Database Date Ranges.

Database	Date Searched	Date Ranges Captured
OVID Medline (R) without revisions (1946 to 1995)	June 23, 2014	1989–1995
OVID Medline (R) without revisions (1996 to June 2014)	June 23, 2014	1995–June 2014
OVID Healthstar (1966 to May 2014)	June 24, 2014	1989–Current
Embase Classic + Embase (1947 to June 2014)	June 24, 2014	1989–Current
CINAHL	June 24, 2014	1989–2014
EBM Reviews – Cochrane Database of Systematic Reviews (2005 to May 2014)	June 24, 2014	2005–Current
PsychINFO (1987 to June 2014)	June 24, 2014	1989–Current
ERIC (1966–Current)	June 26, 2014	1989–Current

**Table 2:** Database Search Terms.

Category	Terms Searched
Acquired Brain Injury	brain injur*
	exp Brain Injuries/
	exp Craniocerebral Trauma/
	exp Brain Concussion/
	exp Head Injuries, Closed/
	acquired brain injury
	exp Brain Neoplasms/
	brain tumor*
	exp Stroke/
	stroke*
	cerebrovascular accident*
	cerebral vascular accident*
Hospital-to-School Transition	transition*
	hospital-to-school
	exp School Health Services/
	exp Rehabilitation/
	(school* adj3 re-entry*)
	(school* adj3 reentry*)
	(school* adj3 re-integrat*)
	(school* adj3 reintegrat*)
	exp adolescent, hospitalized/
	exp child, hospitalized/
	hospitalized child*
	hospital classroom
	school liaison*
	exp Education, Special/
	(return adj3 school*)
	(return adj3 learn*)
	(return adj3 classroom*)
	(classroom adj3 reintegrat*)
	discharge plan*
	exp Patient Discharge/
Age	Limit "all child (0 to 18 years)"
Date Range	Limit to yr = "1989-Current"

Note: All items in each category were combined with Boolean operator “OR”, then categories were combined with Boolean operator “AND”. This table represents the specific search and MeSH terms used on OVID Medline, and adjustments were made for search terms associated with other databases.

*All derivatives of the word.

### Article selection

To select articles for this review, we applied the following inclusion and exclusion criteria. Eligible studies were (1) published in a peer-reviewed journal between 1989 and June 2014; and they involved (2) a clinical population with ABI; (3) a majority sample of children or average sample age of 20 years or younger; (4) an intentional, structured intervention affecting hospital-to-school transitions or components involved in the transition process; (5) an experimental design, entailing a comparison group or multiple baselines for a case study; and (6) a statistically evaluated health outcome (e.g., neuropsychological functioning, social competence, anxiety, depression). We excluded: (1) articles reporting on satisfaction about health services; (2) articles focusing on preschool children or adults; (3) opinion articles; (4) qualitative studies; and (5) articles reporting level IV evidence (based on the American Academy of Neurology classification of evidence) with only pre-and post-intervention analyses, [34]. We applied these criteria to ensure that the best practices and effective components of intervention that we identify are based on the best available evidence.

**Fig 1:**
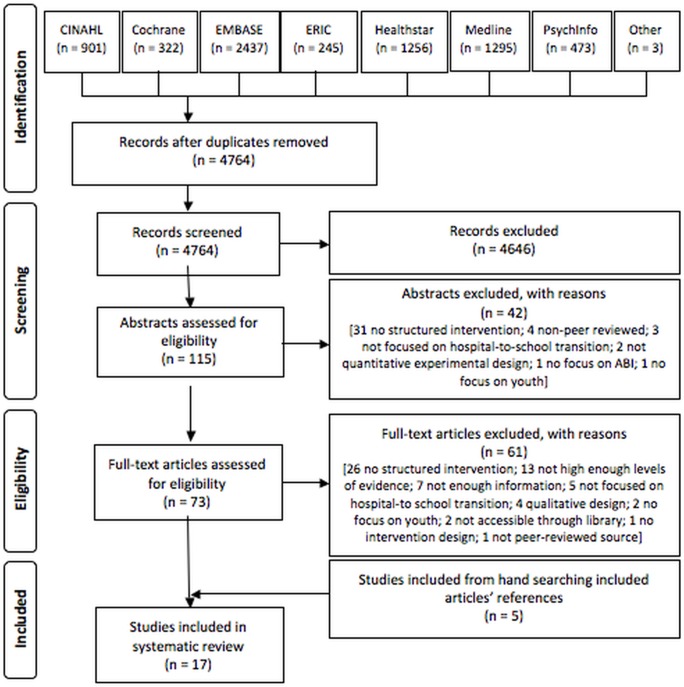
Flow diagram of study identification, screening, eligibility and inclusion (adapted from [63]).

In our initial search, we identified 6938 articles for potential inclusion in this review (Fig 1). We imported the titles and abstracts of those articles into Endnote. The first two authors (SL, LH) independently reviewed them, removing duplicates and an additional 4646 articles that did not meet our inclusion criteria (Fig 1). We read the remaining 73 articles, independently applying the inclusion criteria. We resolved discrepancies of opinion through discussion with the research team. Ultimately, we selected 12 articles from the initial search to include in our final sample. We also included an additional five articles that we identified by manually reviewing the reference lists of other articles selected for inclusion. Throughout this process, we maintained a log of inclusion and exclusion decisions to provide an audit trail.

### Data abstraction and synthesis

One team member (LH) extracted and compiled data from the 17 articles selected for review, using a structured abstraction form based on similar reviews conducted on hospital-to-school transitions for childhood cancer [35] and interventions for self-management of childhood disability [36]. She pilot tested this abstraction form on three articles, refining it further to capture key elements before applying it to all 17 articles. She abstracted information on each study (authorship, year of publication, country, recruitment setting, and experimental design), participant set (recruitment procedures, sample size, gender, population, age, condition severity, and social demographics), intervention (duration, content, format, dosage, and mode of delivery), setting of intervention (hospital, school), method of intervention, type of program facilitator, and outcomes. If data was missing from an article, we contacted the authors for additional information. The first and second authors reviewed all 17 articles and the abstracted data for accuracy. Two additional research team members also reviewed a sample of the articles and abstracted data. We also noted the limitations of each study and risk of bias.

Given the heterogeneity of the studies reviewed (range in study populations, interventions, and outcome measures), it was not feasible to conduct a meta analysis. We synthesized our findings according to the guidelines for narrative synthesis outlined by Petticrew and Roberts [37], which involves a structured interrogation and summary of all studies selected for inclusion. In the first stage of synthesis, the first two authors organized the studies into logical categories to guide our analysis. We grouped studies by levels of evidence [34] and outcomes to enhance our understanding of intervention effectiveness. Then we grouped studies by type of intervention to explore components of different intervention models. In the second stage, we conducted a “within study analysis” by developing a narrative description of each study’s findings and its quality. In the third stage, we conducted a cross-study synthesis to produce a summary of study findings, taking into account variations in study design and quality, representativeness across diverse populations and health care systems. After the first two authors completed their data abstraction and initial analysis, the research team met to consider their findings and resolve any discrepancies through discussion. This method of data abstraction and synthesis is relevant for reviews of studies with diverse methodologies [38].

### Methodological quality assessment

The research team based its findings and recommendations for further research and development of effective hospital-to-school reintegration interventions on the overall strength and quality of the evidence reviewed. As an overall measure of bias, we used the American Academy of Neurology (AAN) classification of evidence for therapeutic intervention, which can help inform evidence-based guidelines and interventions [34]. Using this classificatory system, the first two authors independently reviewed each study, assigning an AAN class and noting significant issues concerning bias. We resolved any discrepancies in their ratings through discussion.

The first two authors also assessed the methodological quality of the studies, using the Physiotherapy Evidence Database (PEDro) scale [39] for seven of the articles reviewed, which reported on RCTs, and a version of the Strengthening of the Reporting of Observational Studies in Epidemiology (STROBE) assessment tool [40] for the remaining ten articles, which reported on pre-post designs [41] (S1 and S2 Tables). We used the PEDro scale to assess 11 quality criteria, awarding each of the seven studies a score of 0 (absent) or 1 (present) for each of the PEDro criterion [39]. We awarded scores ranging from 4–8 points to each study, out of a possible total of 11 points. It should be noted that for practical and ethical reasons, blinding is difficult in therapeutic and rehabilitative intervention processes. In most of the articles reviewed, researchers were unable to adequately conceal and blind conditions in their project—and this negatively affected their PEDro scores on items 3, 5, 6, and 7. Only one study [42] concealed the allocation of participants and blind assessors to the treatment condition, and another study [42] blinded subjects to their treatment allocation. As another common source of lost points, five of the seven articles did not report intention-to-treat analyses of participants.

The first two authors used the STROBE tool to assess the remaining ten studies for 22 quality criteria, awarding a score ranging from 0 (absent) to 1 (present) for each criterion (S2 Table). Any discrepancies in their ratings were resolved through discussion. We awarded scores ranging from 16.5 to 19.6 points to each study, out of a possible total score of 22. Common reasons for loss of points included lack of sample size justification, lack of description of how missing data were addressed, and lack of flow diagrams of participant inclusion. In the case of most studies, several STROBE criteria were found to be inapplicable, including items related to control groups (i.e., justification of controls per case, methods used to examine subgroups and interactions), attrition and missing data, and data related to sensitivity and risk analyses.

We also followed the Preferred Reporting Items for Systematic Reviews (PRISMA), a method of transparent reporting (S3 Table)[41].

## Results

### Study and participant characteristics

Seventeen articles met the inclusion criteria for this review (Table 3). Eleven of the reported studies were conducted in the United States, two in Sweden, and one each in Canada, Brazil, Australia, and China. Eleven of them involved RCT designs, five involved case study experimental designs with multiple baselines and a comparison group, and one involved a single subject design. Sample sizes ranged from 2–72 participants. In fifteen of the studies, the majority of participants had moderate to severe ABI. Two of the studies [43, 44] specifically included youth with brain tumors. The time lapsed since ABI ranged from one month to 13 years. The ages of participants ranged from 4–19 years old. Six of the articles did not report the socio-demographic characteristics of participants. In seven of the remaining studies, the majority of participants were Caucasian. In two of the remaining studies, the majority of participants were from an ethnic minority group [44, 45]. In all cases but one [46] participants included both males and females.

**Table 3:** Overview of Studies.

First Author, Year	Country	Recruit. Setting	Study Design	N (% female)	Socio-demographics	Clinical Population	Time since ABI (mean, SD)	Age range (mean, SD)	Time removed from school	Qlty(AAN class)
Agnihotri, 2014	Canada	Hospital	Longitudinal, multiple descriptive case study	I: 4 (0) C: 1 (100)	n/s	60% severe TBI, 20% moderate TBI; 1 optic glioma and surgeries	4–13 y (8.8, 4.27)	13–16 (14.6, 1.52)	n/s	IV
Beardmore, 1999	Australia	Hospital	Repeated measures, matched groups	I: 11 (27) C: 10 (30)	n/s	Severe TBI	1–5 y (I: 3.55, 1.75; C: 3.20, 1.14)	9–16 (I: 13.27, 2.24; C: 13.3, 2.36)	n/s	IV
Braga, 2005	Brazil	Pediatric rehab clinic	RCT	I: 44* (47) C: 43* (44)	n/s race/ethnicity; Average parent education 11.42y	Moderate or severe TBI	6–30 m (n/s)	5–12 (8.1, 2.47)	n/s	I
Chan, 2011	China	Child assess. center	RCT	I: 16 (31.3) C: 16 (43.8)	n/s	65.6% TBI, 18.7% brain tumor, 15.6% arteriovenous malformation	n/s (I: 3.31, 2.14; C: 3.94, 2.72)	8–18 (12.4. 3.02)	n/s	II
Feeney, 2003	USA	School referral	Single-subject reversal design	2 (50)	100% Caucasian; working parents with high school or more education	Severe TBI	1–2 y (1.5, 0.71)	6–7 (6.5, 0.71)	3 m-2 y	IV
Glang, 1997	USA	Schools	Multiple baseline across subjects	3 (0)	n/s	67% severe TBI, 33% mild TBI	4–6 y (5.0, 1.00)	8–13 (10.7, 2.52)	0–5 m	IV
Glang, 2007	USA	State brain injury assoc.	Randomized trial	31 (94) [breakdown n/s]	74% Caucasian, 13% Hispanic / Latino, 3% African American, 3% Asian American, 3% other; Parents: 48% some college or specialized training, 36% college graduate, 10% high school, 6% graduate degree	Child with brain injury (intervention targeting parents)	1–9 y (n/s)	4–17 (n/s)	n/s	I
Kesler, 2011	USA	n/s	One-arm open randomized pilot trial	T1: 23 (39) T2: 17 (n/s)	52% classified minority status; average maternal education 17.7y	History of brain malignancy, 56% acute lymphoblastic leukemia, 44% posterior fossa brain tumors	6–126 m (37, 30)	7–19 (12.6, 4.1)	n/s	II
Mottram, 2004	USA	Institute for child dev.	Multiple baseline across subjects with comparison groups	I: 3 (0) Comparison: 3 (67) Control: 2 (50)	62.5% African American, 25.0% Hispanic, 12.5% Caucasian/Philippine	I: TBI, hydrocephalus, encephalopathy; Comparison: hydrocephalus, spina bifida, cerebral palsy; Control: Nonnan’s Syndrome, peripheral neuropathy	<1–7 y (n/s)	7.9–12.4 (9.4, 2.43)	n/s	IV
Suzman, 1997	USA	Child transition program	Case study	5 (40)	100% African American	80% TBI due to motor vehicle accident, 20% brain hemorrhage secondary to arteriovenous malformation	3–9 m (n/s)	6–11 (8.2, 1.92)	n/s	IV
van‘t Hooft, 2005	Sweden	Hospital	RCT	I: 18 (33) C: 20 (50)	n/s	34% GCS>8, 21% GCS<8, 5% encephalitis, 3% anoxia, 37% brain malignancies	1–5 y (I: 2.2 1.0; C: 2.6 1.2)	9–17 (I: 11.7, 2.3; C: 12.6, 2.6)	n/s	I
van‘t Hooft, 2007	Sweden	Hospital	RCT	I: 18 (33) C: 20 (50)	n/s	34% GCS>8, 21% GCS<8, 5% encephalitis, 3% anoxia, 37% brain malignancies	1–5 y (I: 2.2, 1.0; C: 2.6, 1.2)	9–17 (I: 11.7, 2.3; C: 12.05, 2.6)	n/s	I
Wade, 2006a	USA	Hospital	RCT	I: 20 (45) C: 20 (40)	75% Caucasian, 25% African American; Parents: 50% married; 45.4% more education than high school	Moderate to severe TBI	1–24 m (I: 8.73, 4.34; C: 8.83, 4.85)	5–16 (I: 10.94, 2.62; C: 10.72, 3.31)	n/s	I
Wade, 2006b	USA	Hospital	RCT	I: 16 (38) C: 16 (31)	81% Caucasian, 19% African American	67.6% moderate TBI (GCS 9–12), 32.4% severe TBI (GCS<8)	1–24 m; (I: 13.48, 6.86; C: 14.05, 7.54)	5–16 (I: 10.92, 2.45; C: 11.0, 3.93)	n/s	I
Wade, 2008	USA	Hospital	Randomized trial	Audio condition: 5 (n/s) Non-audio condition: 4 (n/s) (total 44% female)	11% African American, 11% biracial, 78% n/s; Parents: 44% married and living together; SES varied	22.2% moderate TBI (GCS 9–12), 77.8% severe TBI (GCS<8)	2–20 m; (9.33, n/s)	11–18 (15.04, n/s)	n/s	III
Wade, 2010	USA	Hospital	RCT	I: 16 (62) C: 19 (42)	91% Caucasian, 9% n/s	I: TBI, mean GCS 9.47C: TBI, mean GCS 10.5	≤18 m (I: 8.75, 5.51; C: 10.32, 4.42)	11–18 (I: 14.02, 2.45C: 14.49, 2.13)	n/s	II
Wade, 2011	USA	Hospital	RCT	I: 16 (62) C: 19 (42)	91% Caucasian, 9% n/s; mean income $35,000-$39,999	I: TBI, mean GCS 9.47 C: TBI, mean CGS 10.5	≤18 m (I: 8.75, 5.51; C: 10.32, 4.42)	11–18 (I: 14.02, 2.45C: 14.49, 2.13)	n/s	II

I = intervention, C = control, RCT = Randomized controlled trial;

AAN Classes[64]: I = rigorous RCT; II = matched prospective cohort studies or RCTs in a representative population lacking one of the criteria in class I; III = all other controlled trials; IV, all other studies

*sample size at initial enrolment; final sample of 38 intervention and 34 control participants;

**sample size at initial enrolment; final sample of 71 participants;

***sample had majority of participants with TBI and was therefore included in this review.

### Types of intervention

The articles reviewed investigated several different types of intervention (S4 Table), including arts-based activities [47], problem-solving activities [24], clinician-led information sessions [48], behavioral and cognitive interventions [42, 49], family or social support interventions [23, 46, 50–52], online interventions [44, 53–57], and multi-component interventions [45, 58]. In Agnihoti et al. [47], professional theatre artists used an arts-based approach to deliver a social skills intervention in a hospital-based classroom. The intervention focused on youth aged 13–16 years old with moderate to severe ABI. The program was implemented through 20 group sessions, lasting 4 hours each, with participants meeting daily over a period of 4 weeks. It emphasized voice work, breathing, movement, physical warm-up, character development, script analysis, writing skills, three-dimensional awareness, group dynamics, story development, mask work, and clowning.

In Beardmore’s [48] study, clinicians led information sessions (“injury information group”) in participants’ homes. The intervention was for youth aged 9–16 years old with severe ABI [48]. It was implemented through two individual sessions, delivered over the course of one month. It also incorporated parental involvement. Clinicians helped participants construct a timeline of events about their accident, including details about the timing and location of the accident, information about their time in hospital, rehabilitation, and home, and information about timing and location of return-to-school. Throughout the process, researchers highlighted points that were particularly relevant to each participant. They also provided additional information about the brain and ABI processes to participants.

In Braga [23], clinicians implemented a family-supported intervention in participants’ homes. The intervention was designed for children aged 5–12 years old with moderate or severe ABI. It was implemented through daily one-on-one sessions over the course of a year. It also incorporated practice homework and parental involvement. Children selected and practiced activities from an illustrated guide, creating individualized rehabilitation routines and an illustrated manual. Parents observed and took increasing responsibility for their child’s rehabilitation routines, and they attended information sessions and support groups.

In Chan and Fong’s [24] study, an occupational therapist used a problem solving skills training approach to provide metacognition training. The intervention was used with children and youth aged 8–18 years old with ABI or a brain tumor. It was implemented through 14 group sessions, lasting three hours each, over seven weeks. Parents also received practice homework assignments.

In Feeney and Ylvisaker’s study [50], teachers and instructional assistants implemented a support-oriented behavioral and cognitive intervention in a school setting. The intervention focused on self-regulation among children aged 6–7 years old with severe ABI. It was implemented daily, over the course of one month. It involved student observation of performance and behavior, and components included daily routines, positive momentum, reducing errors, escape communication, adult communication style, photo cues, and reviewing goals.

In Wade’s study [53], a clinical psychologist implemented a “family problem solving” program. The intervention was designed for children (average age of 10.8 years old) with a moderate or severe ABI and their families. It was implemented through 7–11 family sessions, lasting 75–100 minutes each, which took place every other week over six-months. The therapist presented didactic information on a range of ABI-related topics and helped participants problem solve, using a five-step process (aim, brainstorm, choose, do it, evaluate).

In Van‘t Hooft et al. [42, 49], a teacher or parent delivered Amsterdam Memory and Attention Training for Children (AMAT-c) and Swedish Memory and Attention ReTraining (SMART) on a one-on-one basis. The intervention took place in a hospital setting. It focused on children and youth aged 9–17 with moderate to severe ABI or brain malignancies. It was implemented through 119 half-hour sessions over the course of 17 weeks.

In Glang’s [46] study, friendship facilitators used a friendship-building process to facilitate social integration. This intervention was applied children aged 8–13 years old with mild or severe ABI, family members, peers, and teachers. It was implemented through 3–9 group sessions over the course of three months. A friendship facilitator led group sessions to introduce the intervention and made weekly phone contact with parents to gauge the current social contacts. Facilitators then implemented the four-phase friendship-building process, which included: (1) gathering information through interviews with the student, parent, school staff, and peers; (2) recruiting family members, school staff, and peers to be team members; (3) conducting an initial team meeting to share information and create visions for the future; and (4) holding review meetings every 2–3 weeks to revise goals and strategies and review team membership and responsibilities.

We also reviewed several studies on web-based interventions aiming to improve transitions from hospital to home or school for youth with ABI. In Glang [51], parents received a CD-ROM-based advocacy training resource to provide communication skills training and peer support for families of children with ABI. This intervention was aimed at children and youth aged 4–17 year olds with ABI and their parents. It was delivered through one 1-hour-long CD-ROM session at home.

In Kesler [44], a web-based cognitive rehabilitation curriculum program was delivered to children and youth at home. This program was for children and youth age 7–19 years old with brain malignancy. It was implemented through 40 daily sessions, lasting 20 minutes each, over the course of eight weeks. Each session consisted of six tasks related to working memory, attention, and cognitive flexibility. Exercises were adapted based on participants’ performance. The intervention also incorporated a parental component.

In Wade [54], a therapist used video conferencing technology to deliver an online version of the family problem solving intervention described above. It involved youth aged 11–18 years old with moderate to severe ABI, parents, and siblings. It was implemented online, through eight core sessions and an additional six sessions addressing topics related to family functioning. Families participated from home.

In Wade [55], a staff psychologist delivered another online program, the “Teen Online Problem Solving” intervention, which could be used by multiple family members at the same time. This intervention was for youth aged 11–18 years old with moderate or severe ABI and their family members. It was implemented through 10–16 online sessions, designed to enhance executive function and social skills essential for successful adult functioning, with an emphasis on self-monitoring. Wade’s research group subsequently ran the intervention again, among participants with an average age of 14 years old [55] and 15 years old [56].

Two of the studies examined interventions entailing a multi-component approach. In Mottram and Berger-Gross [58], children earned tokens for following pre-determined behavioral rules for set periods of time in a hospital-based-school. If they received enough tokens in a day, they gained access to a mystery motivator. The number of tokens needed to access daily mystery motivators increased every three consecutive days that participants accessed mystery motivators. This intervention was used with children aged 7–12 years old with ABI. It was implemented through approximately 50 sessions, lasting 1 hour each, over 10 weeks.

In Suzman [45], a researcher implemented a multi-component cognitive-behavioral intervention involving computerized problem-solving tasks in a school setting. This intervention involved children aged 6–11 years old with moderate to severe ABI or brain hemorrhage. It was implemented through 22–26 sessions, lasting 40 minutes each, over a period of 8–9 weeks. The researcher delivered sequential training sessions on self-instruction, self-regulation, metacognition, and attribution, introducing a new training session after participants had mastered the previous sessions twice in a row, with the help of reinforcement.

### Outcomes and study findings

Outcome measures varied greatly across the studies reviewed (S5 Table). They included a variety of standardized and non-standardized measures assessing social skills, participation, attention/memory, adjustment/coping strategies, knowledge of ABI, self-esteem, self-efficacy, behavior, cognitive functioning, physical and motor functioning, non-verbal abstract reasoning, and problem-solving. Secondary measures addressed outcomes including parental psychological distress, parent-adolescent interactions, perception of parental advocacy effectiveness, family burden, and family functioning.

Most studies used Cohen’s d, where effect size of 0.2 is considered to represent a small effect, 0.5 a medium effect and >0.8 a large effect [59]. When authors did not report an effect size, we calculated it (where possible) using the means and standard deviations to report Cohen’s d [59]. Two of the studies reported significant improvements in knowledge of ABI (see S3 Table). One reported a medium effect size (d = 0.76) [48] and the other a small effect (d = 0.28) [51]. Seven studies reported significant improvements in cognitive functioning, where effect sizes ranged from small to large. For example, Braga et al. [23] found a small effect (d = 0.3) [23] using the Wechsler Intelligence Scale for Children, while Kessler et al. [44] found a large effect (d = 1.0) using the same scale. Other measures for cognitive functioning included abstract reasoning (large effect, d = 1.9) [24], metacognition (nature, planning, representing, monitoring metacomponent correctness) (large effects in two studies) [43, 56], executive functioning (large effect) [56], and sustained selective attention and memory [42, 49] (effect size not specified).

Several studies reported improvements in social functioning, including improvements in social contacts (effect size not specified) [46], interpersonal negotiation strategies (large effect, d = 1.58) [24], and problem solving in social situations (large effect, d = 1.58) [24, 45]. Two studies reported significant improvements in behavior, including improvements in externalizing and internalizing symptoms (small and medium effects) [56] and reduced parent-youth conflict (medium effect) [55].

Three studies reported improvements in psychological functioning, including improved child adjustment (internalizing symptoms and decreased anxiety/depressive symptoms and withdrawal) [53, 58], (with effect sizes ranging from d = 0.18–0.58, small to medium effect) and a large effect in behavioral observation [58–59].

In regards to secondary measures, one study found significant improvements on the Canadian Occupational Performance Measure (COPM) from child and parent perspectives (both large effects) [24]. Other studies reported a small effect in self-esteem [48] and improved physical and motor functioning (medium effect) [23]. One of the reported secondary outcomes entailed significant improvements in perceived effectiveness of parent advocates [51], with a medium effect (d = 0.52). It is important to note that several studies did not report effect size and/or provide sufficient information to calculate it [45, 46, 47, 49, 50] and thus, these studies should be interpreted with caution.

In regards to their rigor, we classified six studies as level I (rigorous RCT), four studies as level II (matched cohort studies or RCT in a representative population lacking one criterion in level I), one study as level III (all other control trials), and the remainder as level IV [34]. In studies that reported effect sizes, they ranged from small to large (d = 0.28 to 1.0). It was interesting to note that fewer studies with level IV evidence reported significant outcomes.

### Components of the interventions

The reported interventions varied greatly in length, duration, number of sessions, and delivery format. The number of sessions ranged from 1–119, covering a period from one day to one year (S6 Table). The estimated total intervention time ranged from 1–80 hours. Eight of the interventions were delivered by a clinician [23–24, 48, 53–58] four by a teacher or educator [42, 46, 49–50], one by a professional theatre artist [47], one by a researcher [45], one by a multi-disciplinary team [58], one via CD-ROM [51], and one through an online program [44]. Five of the interventions were delivered online [54–56], four in multiple settings [23, 42, 49, 53], three in participant homes [44, 46, 48], three in schools [20, 45, 50], and two in a hospital or a classroom in a hospital-based school [5, 58]. One of the interventions involved phone contact [20]. One study did not specify the delivery setting or medium [24].

Only three interventions were group-based [5, 24, 46], and the remaining interventions were one-on-one. Five of the interventions were individualized [23, 42, 46, 49, 58], four were standardized [24, 44, 47, 51], and eight entailed a combination of both approaches [45, 48, 50, 53–58]. Twelve of the interventions involved meetings, thirteen involved homework exercises, two involved a parental component, five engaged other family members (siblings), three engaged teachers, and one included a peer component.

## Discussion and Conclusions

To our knowledge, this is the first systematic review to undertake a comprehensive synthesis of pediatric hospital-to-school interventions focused on children and/or youth with ABI. We examined interventions that aimed to support pediatric participants’ return to school environments following a brain injury. Given the incidence of ABI among school-age populations, many educators are likely to encounter a child with a brain injury. The high prevalence of ABI in the school population is demonstrated by a recent survey of 9000 students in Ontario, Canada, which found that as many as 20 percent of adolescents report having experienced a brain injury in their lifetime [60]. Our focus on children and youth with ABI is salient given recent health system efforts to use models of collaborative care and evidence-based guidelines to improve efficiency, quality of service, and patient outcomes, while encouraging cost reductions [54–55]. Identification of best practices in pediatric hospital-to-school transitions can help increase cross-sector collaboration among clinicians, decision-makers, and educators to help implement cost-effective and efficient hospital-to-school reintegration interventions and ultimately improve health outcomes for youth with ABI.

Our findings suggest that school reintegration interventions for youth with moderate or severe ABI have the potential to improve knowledge of ABI, cognitive functioning, behavior, problem solving, social skills, and coping. However, it is important to note that the strength of evidence of each outcome varied. In contrast to other reviews of school reintegration for children with cancer [35], the interventions included in this review focused primarily on individual behavior and cognitive functioning—and less on increasing knowledge of the condition and family support. Prevatt et al. [35] also found that hospital-to-school reintegration interventions primarily focused on educating school personnel, providing peer education and comprehensive programs, and connecting hospitals, clinicians, families, and youth; in contrast, our findings demonstrate very little, if any, emphasis on educating teachers or peers. Educating teachers on ABI is essential, given the findings of Ilie and colleagues [60] that one in five students in grades 7–12 report a brain injury. Moreover, since some ABI-related challenges may not emerge until adolescence, there is an increased risk of educators overlooking the effects of an ABI if it has been many years since a child or youth sustained the injury. It is important to monitor students with ABI over time, especially since many educators do not understand the emerging nature of deficits in childhood ABI.

Incorporating a teacher and peer component into school reintegration interventions is also critical because youth with chronic conditions or injuries often experience stigma, bullying, rejection from peers, and depression [35, 60]. A recent Ontario study showed that students with ABI are twice as likely to report elevated psychological distress and to be prescribed anxiety/depression medication compared to students without an ABI [17]. Students who reported previous ABI were also three times more likely to attempt suicide and roughly twice as likely to be bullied compared to those who had not experienced an ABI. The study also found adolescents who had experienced a brain injury are more likely to become bullies themselves, use alcohol or cannabis, and engage in antisocial behaviors. In this context, peer support at school is a strong predictor of positive psychological adaptation for youth with a chronic condition [35]. Providing more education and knowledge about a youth’s condition to educators and peers can help increase support and reduce isolation and bullying [61]. Given the strong relationship between self-reported brain injury and mental health problems, focused education for school personnel and screening for mental health problems in adolescents with ABI is clearly warranted.

To meet the academic, medical, social, emotional, and behavioral needs of youth, multidisciplinary collaboration among clinicians, educators, family, and youth is crucial. The effects of ABI are complex and affect several areas of functioning; as such, interventions should address the multifaceted needs of youth as they return to school [61]. Some researchers argue that given the changing level of functioning among youth with ABI, it is essential to provide a variety of flexible and coordinated support services [62]. Although most of the interventions addressed in this review were flexible in terms of adapting to participants’ individual needs, very few of them coordinated services between hospitals, clinicians, and schools. We did find evidence of several comprehensive hospital-to-school programs in our search; however, we have not included them in this review because most were program descriptions that lacked evaluated outcomes.

Our findings suggest that effective hospital-to-school reintegration interventions may involve several different components. Common components of successful interventions included one-on-one sessions led by a trained clinician or educator, homework activities, and parental involvement. These findings are consistent with previous research on social integration of people with ABI [47]. Other multi-media methods and materials (e.g., videos, art, games, and role playing) may also be worth exploring as strategies to help youth transition back to school. Younger children may also benefit from storytelling or the use of puppets [61], while older youth may find the support of peer mentors advantageous. Further work is needed to explore the effectiveness of different types of interventions.

Given that adolescents with moderate to severe ABI have a high likelihood of experiencing mental health and behavioral problems, it is essential for educators, mental health professionals, and rehabilitation professionals to work collaboratively and proactively together to ensure vigilant screening, ongoing monitoring, and early intervention to mitigate the negative effects of brain injury. We were surprised that most of the interventions occurred years after participants had originally sustained a brain injury, and the programs seemed more reactive than preventative in nature. We would recommend that future interventions implement educational and support components earlier on, particularly when youth are seen in the hospital. It is also important to consider different phases of the adolescent lifespan, including transitions to high school, post-secondary education, and employment. Taking a preventative approach to mental health and behavioral problems is important because such issues are linked to the psychosocial phases of recovery (i.e., initial phase versus later phase) that are common across a range of medical diagnoses (e.g., academic, cognitive and social-emotional challenges) [17, 35].

### Key Messages

- A holistic approach to rehabilitation (addressing behavioral, physical, cognitive, social and emotional needs) is proposed for students with mild, moderate and severe ABI who are transitioning from hospital to school.
- Interventions that significantly improved youth’s and educators’ knowledge of ABI were delivered in the home, used a one-to-one format, involved a practice component, had parent involvement, and ranged from one to two sessions.
- Interventions that improved cognitive functioning commonly took place in the home, were delivered one-to-one in-person or online, had homework components, parent and/or teacher involvement, and ran from seven weeks to one year.
- Interventions that had a significant impact on social functioning commonly took place at school or online at home, were delivered in a one-to-one format, were structured rather than individualized, involved meetings and homework, had parent involvement, and ranged in duration from 7–16 weeks.
- Interventions that significantly affected psychological functioning were delivered in a variety of settings, had a one-to-one format, involved an individualized component, had parent and/or sibling involvement, and ranged from 10 to 24 weeks.

### Limitations

This review involves several limitations that should be considered. First, the specific databases and search terms that we selected for our search strategy may have limited our ability to find relevant publications. However, we designed our search strategy in consultation with an experienced librarian and experts in the field. Second, we only included published, peer-reviewed articles in this review. Future reviews should consider including grey literature.

We also identified several limitations in the studies we reviewed that future research should address. First, many of the studies entailed small and heterogeneous samples. Second, they used a variety of unstandardized and standardized outcome measures, which limited our ability to compare effectiveness across studies and interventions. Third, some interventions were tailored to individual participants—and it is difficult to know if they have would have the same effects if applied to different sample sets. Fourth, some studies entailed convenience sampling and thus, may have engaged participants who were more motivated than others to recover. Fifth, many studies examined interventions involving several components, and it is unclear which component(s) made an impact. Sixth, few of the interventions and studies were based on theoretical frameworks, which can critically inform components of interventions and the measures used to evaluate them. Seventh, time away from academia was only reported in two of the 17 studies [46, 50]. The effects of the injury as compared to the effects of missing class time can therefore not be measured. Finally, several of the studies included in this review seemed to report on the same two interventions; thus, the overall strength of the evidence in this review should be interpreted with caution.

### Future research and interventions

While we found some evidence to suggest that cognitive, behavioral, and social functioning interventions have the potential to improve school reintegration processes for youth with ABI, we have also identified key areas for future research and further development of hospital-to-school reintegration interventions. First, more research is needed to tease out differences in hospital-to-school transition experiences among youth with traumatic brain injury, including age, gender, cultural, geographic (rural/urban), and socio-economic differences. Second, more research is needed to explore differences in hospital-to-school reintegration outcomes by type of school (e.g., public, private). Third, of the articles reviewed there was little consideration granted to how the cause and severity of ABIs may affect transitions back to school. Mild ABI (i.e., concussions) was notably absent from the literature and deserves attention in future interventions. Fourth, there is a critical need for more comprehensive interventions that link clinicians, educators, families, and youth. Fifth, in regards to methodological design, future interventions and evaluations should consider enacting longer-term follow-up periods, larger samples sizes, and more rigorous designs with standardized measures. Finally, future studies should consider measuring peer integration, which is a good indicator of a successful transition. It would also be helpful to have more standardized measures on health outcomes and quality of life.

## Supporting Information

S1 Table PEDro Scores: ABI hospital-to-school intervention review. (XLSX)

S2 Table STROBE Scores: ABI hospital-to-school intervention review. (XLSX)

S3 Table PRISMA checklist. (DOC)

S4 Table Intervention types and limitations. (XLSX)

S5 Table Intervention outcomes. (XLSX)

S6 Table Intervention components. (XLS)
